# Acceptance level and influencing factors of advance care planning among patients with schizophrenia in China: a cross-sectional survey

**DOI:** 10.3389/fpsyt.2025.1652534

**Published:** 2025-09-08

**Authors:** Shicong Liang, Lei Huang, Shu-Tong Tang, Hong-Xing Li, Jia-Wei Huang, Wen-Qing Zhou, Zhi-Chun Xia

**Affiliations:** ^1^ The Affiliated Brain Hospital, Guangzhou Medical University, Guangzhou, China; ^2^ Key Laboratory of Neurogenetics and Channelopathies of Guangdong Province and the Ministry of Education of China, Guangzhou Medical University, Guangzhou, China; ^3^ School of Nursing, Guangzhou Medical University, Guangzhou, China; ^4^ The Seventh Affiliated Hospital of Sun Yat-sen Universit, Shenzhen, China

**Keywords:** Advance Care Planning, schizophrenia, cross-sectional, influencing factors, acceptance level

## Abstract

**Objective:**

This study aimed to explore the current acceptance of advance care planning (ACP) among patients with schizophrenia and investigate the influencing factors.

**Methods:**

A cross-sectional study was conducted from September 2023 to January 2024 using a convenience sampling method. A total of 225 patients with schizophrenia were selected from a Grade A, Class III Psychiatric Hospital and the Department of Psychiatry of a Grade A, Class III General Hospital in Guangzhou, China. The Advance Care Planning Readiness Scale, the Chinese version of the Brief Neurocognitive Test Battery(C-BCT), the Brief Psychiatric Rating Scale(BPRS), and the Medication Adherence Questionnaire(MAQ) were used to survey the patients with schizophrenia.

**Results:**

Mean ACP readiness score was 84.76 ± 11.97, with 55.6% showing high acceptance. Readiness positively correlated with age (r=0.161, p=0.021) and hospitalization frequency (r=0.235, p=0.001), and negatively with symptom severity (r=-0.159, p=0.022). Patients with comorbidities (p=0.001) or personal/family health crises (p=0.033) had higher readiness. Regression analysis identified cognitive impairment, religious beliefs, hospitalization frequency, and medication adherence as key predictors (R²=0.118, p<0.001), explaining 11.8% of variance.

**Conclusions:**

Schizophrenia patients demonstrate higher than average ACP acceptance, with 55.6% of the patients showing a high level of acceptance. Improved medication adherence, frequent hospitalizations, religious beliefs and reduced thinking disorder severity. Culturally sensitive health education is recommended to facilitate ACP discussions in clinical practice.

## 1 Introduction

Schizophrenia is a severe chronic mental disorder (1), resulting from genetic and environmental interactions, ranking among the top ten most disabling diseases globally and representing the fourth leading cause of disability worldwide (2, 3). Long-term antipsychotics can stabilize symptoms but rarely restore full autonomy (4, 5). Sampogna et al. (6) confirms that quality of life represents a central element for selecting the appropriate treatment for people with schizophrenia, However, despite available treatments, the quality of life reported by patients with schizophrenia taking antipsychotics is still very poor. The burden caused by diseases ranks 20th (7) among 369 diseases and injuries worldwide in 2019, and is one of the main causes of disability among young adults (8). Nevertheless, individuals diagnosed with schizophrenia have a 50% lower probability of receiving hospice care compared to other populations (9). Additionally, they frequently do not obtain suitable palliative care (9, 10) and may even undergo unnecessary invasive treatments toward the end of their lives, resulting in a concerning case of overtreatment. Even during the early stages of schizophrenia, the majority of patients still possess some levels of knowledge, judgment, and expression ability, and their legal competence cannot be disregarded. The disease causes a transition in their cognitive abilities, specifically between decision-making and decision-making (11). Consequently, individuals are unable to articulate their treatment preferences and their physical and psychological demands after they lose their decision-making capacity. It has exacerbated the occurrence of involuntary treatment and early death, flagrantly infringed upon the patients’ autonomy in treatment and care, and compromised their dignity and quality of life (12).

Shenzhen became the first region in China to officially implement living wills, also known as ‘Advance Directives (ADs)’ on January 1, 2023 (13). The demand for patient-centered personalized medical services grew with global population aging, particularly among younger patients. Consequently, there has been a significant increase in the discussion surrounding palliative care in China. One specific area of focus is the development of documents about ACP, which outline living wills and have become a prominent topic of research. The fields of medicine, law, and governance have attracted more interest from professionals (14).

Advance Care Planning (ACP) is a relatively new form of palliative care applicable to individuals of any age or health condition. It involves discussions between patients, families, and medical professionals regarding values, life goals, and future medical care (15). In developed nations, ACP is widely used, but in China, its implementation remains limited due to cultural and systemic differences (16). In addition, the majority of domestic research on ACP consists of descriptive studies that focus exclusively on cancer patients (17). There is a scarcity of studies examining ACP in patients with schizophrenia.

For patients and families affected by serious mental disorders such as schizophrenia, significant medical expenses and valuable end-of-life time are often invested, yet the quality of life for these patients remains unassured. Palliative care guidelines recommend that (18) individuals with incurable diseases should prioritize decisions regarding end-of-life treatment. Irwin KE et al. (19) found that the majority of patients with schizophrenia retain the capacity to make informed medical decisions, particularly when they seek assistance from trusted friends or family members. In Irish psychiatric hospitals, 11% (20) of patients are admitted involuntarily, whereas in China, this figure rises dramatically to 60% (21) for patients with mental disorders. Furthermore, patients with schizophrenia exhibit a greater aversion to the inhumane treatment and loss of self-control associated with involuntary hospitalization. This aversion exceeds their dislike of forced treatment (12). Additionally, involuntary treatment may compromise the effectiveness of care and adversely affect patients’ self-esteem. In order to ensure the autonomy of patients as much as possible, the structure and organization of mental health care in Italy developed an innovative nursing model (22), shifting from the asylum-based system to the community-based model, with the gradual closure of all mental hospitals.

Given the Chinese cultural and medical context, it is imperative to research the acceptance of ACP among patients with schizophrenia. This research aims to describe the level of acceptance of ACP among schizophrenia patients and analyze the factors that influence their acceptance. The findings will guide healthcare professionals in providing appropriate ACP health education to patients. Furthermore, it will serve as a solid foundation for the successful implementation, promotion, and research of ACP in China.

## 2 Methods

The subjects of the study were individuals diagnosed with schizophrenia. A convenience sampling method (23) was used to recruit patients diagnosed with schizophrenia admitted to the Department of Psychiatry at a Grade III, Class A psychiatric hospital and a Grade III, Class A general hospital in Guangzhou between September 2023 and January 2024.The inclusion criteria for this study are as follows: (1) meeting the diagnostic criteria for schizophrenia according to the tenth revision of the International Statistical Classification of Diseases and Related Health Problems (ICD-10) (24); (2) being 18 years of age or older; (3) having clear consciousness, communication skills, and the ability to cooperate with the investigation; (4) regularly using antipsychotic drugs for a minimum of 6 months (25), during the maintenance treatment period, with a stable current condition indicated by a Brief Psychiatric Rating Scale (BPRS) (26) total score of less than 35; (5) being fully informed about the study’s content, willing to participate, and providing signed informed consent. The exclusion criteria are as follows: (1) Participants with comorbid mental disorders as defined by ICD-10, including mental retardation, dementia, and psychoactive substance dependence; (2) Participants who were in the acute stage of severe physical illnesses or critical condition requiring constant monitoring during the investigation; (3) Participants with visual and hearing impairments that prevented them from completing the test. The elimination criteria are as follow: (1) Participants with severe neurocognitive impairment as indicated by a T score ≤19 and Deficit = 5 on the Chinese Brief Cognitive Test (C-BCT); Cognitive dysfunction is one of the core features of schizophrenia, affecting cognitive function in approximately 85% of patients (27). The level of cognitive function affects decision-making ability (28), causing patients to lose their ability to identify their own illness and affecting their acceptance of ACP implementation. For individuals with severe cognitive impairment, their decision-making ability is significantly impaired. Therefore, the observed variables in this study need to include cognitive impairment and exclude individuals with severe cognitive impairment. (2) Individuals who experienced stress events and exhibited stress reactions during the investigation, such as restlessness and crying, were deemed unsuitable to continue with the investigation.

### 2.1 Sample size estimation

(1) The acceptance status survey was conducted using a cross-sectional study of quantitative data to estimate the sample size formula: n=Za2×∨ϵ2 . In this study, the significance level *α*=0.05, then Z0.052 =1.96. Based on literature data, the allowable error ∨≈ 0.06, and the relative error *ϵ*=0.1, resulting in a sample size of 138 cases. Considering a 10% dropout rate, the sample size is calculated to be at least 153 cases.

(2) The estimated sample size based on the study of influencing factors should be 5–10 times that of the observed variables. The independent variables in this study are 18, with an estimated minimum sample size: 18x5 = 90, and an estimated maximum sample size:18x10 = 180. In order to minimize errors caused by insufficient sample size, this study collected 180 cases, taking into account a 10% dropout rate, and further expanded the sample size to 200 cases.

In summary, this study estimated the sample size based on the analysis of influencing factors and selected at least 200 patients with schizophrenia for investigation.

### 2.2 Data collection

The patients underwent an initial screening process using the medical record system. Psychiatrists and investigators then picked individuals who met the specific criteria for inclusion and exclusion. Investigators explained the study’s purpose, importance, and procedures to eligible patients. Patients were notified of their prerogative to either engage or discontinue their involvement in the study of their own accord. Before any procedures, patients and/or their families provided informed consent. The patients themselves typically completed the self-assessment. If patients were unable to complete the questionnaire due to factors such as their level of education, the investigators would assist them by reading and providing a detailed explanation of the questionnaire’s contents.

### 2.3 Measures

(1) Form for Collecting General Demographic Data: The study involved extensive consultation of psychiatric data and referenced several elements that influence general data on ACP in both domestic and foreign studies. A self-designed questionnaire was utilized to gather general information on the research subjects, encompassing their gender, age, occupation, marital status, number of children, religious affiliation, educational attainment, monthly income per person in their family, and medical expenditures.

(2) Table of pertinent disease information: Extensive psychiatric data were consulted to determine the characteristics of schizophrenia. Both domestic and foreign studies on the factors influencing disease information on ACP were referenced, as well as the content of the previous End-of-Life experiences scale (29). The study utilized a self-created table to gather pertinent disease information of the participants. This included the duration of illness, frequency of hospitalizations, presence of other medical conditions, physical impairments, personal accidents, experiences of illness among close family members, and instances of involuntary treatment.

(3) ACP Readiness Scale: The scale created by Wang Xinlu (30) in 2019 is primarily used to assess the level of acceptance of ACP by individuals. It serves as a significant instrument for predicting whether an individual will embrace ACP in the future. The assessment comprises elements of attitude, belief, and motivation, totaling 22 items. The Likert 5-point rating system is employed, where a higher total score corresponds to a greater level of ACP acceptance. Simultaneously, ACP acceptance scores can be categorized into four levels: The range from 22 to 43 is classified as the low level, the range from 44 to 65 is classified as the lower middle level, the range from 66 to 87 is classified as the upper middle level, and the range from 88 to 110 is classified as the high level. The Cronbach’s α coefficient for the scale was 0.923.

(4)The Chinese version of the Brief Neurocognitive Test Battery (C-BCT) is an electronic set of simplified neurocognitive tests designed for Chinese patients with schizophrenia (31). Compared with the Mini mental State Examination (MMSE) and the Measurement and Treatment Research to Improve Cognition In Schizophrenia (MCCB), C-BCT is more in line with Chinese characteristics. It can efficiently, accurately, and specifically assess the neurocognitive abilities of Chinese patients across various dimensions (32). Four neuropsychological tests, namely the trail creation test, symbol coding test, continuous performance test, and digit span test, were conducted. The reliability and validity of C-BCT were assessed, revealing a Cronbach’s α coefficient of 0.75.

(5) The Brief Psychiatric Rating Scale (26) (BPRS) is employed to evaluate the intensity of psychopathological symptoms in patients, particularly those diagnosed with schizophrenia. It of the extent to which the condition affects the individual. A Likert scale ranging from 1 to 7 was employed, where a larger cumulative score signifies a greater level of psychopathology. Typically, a clinical criterion of 35 was employed as the overall score. There were a total of 5 components included in the analysis. These factors included negative symptoms (anxiety, sadness, lack of energy) and positive symptoms (hostility and suspicion, activation, thinking problems).The BPRS has demonstrated significant reliability and validity, shown by a Cronbach’s α coefficient ranging from 0.85 to 0.99.

(6) The Morisky Medication Adherence Questionnaire (MAQ) (33), a self-rating scale, was utilized to assess medication issues in individuals diagnosed with schizophrenia. It encompasses various characteristics, including pharmaceutical non-adherence due to forgetfulness, lack of attention, and self-discontinuation. Greater drug compliance is associated with higher scores. The MAQ questionnaire indicates strong reliability and validity, with a Cronbach’s α coefficient of 0.835.

### 2.4 Analysis

Two individuals input the data and verified it. The data analysis was conducted using SPSS 22.0 statistical software. The count statistics were presented as the number of occurrences and the corresponding percentage. The measurement data were characterized using the mean value plus or minus the standard deviation, and the correlation analysis was conducted using Spearman’s correlation analysis. The Mann-Whitney U and Kruskal-Wallis H rank sum tests were employed for univariate analysis. The study employed multivariate linear regression analysis to conduct a multivariate analysis. A test standard of 0.05 was used, and statistical significance was determined by a P-value of less than 0.05.

### 2.5 Quality control

The study subjects were selected in strict accordance with the inclusion and exclusion criteria, established a good relationship with the patients, used plain language, fully obtained the consent of the patients to participate in the investigation, timely answered the patients’ questions and understanding deviations, and guided the patients to correctly understand ACP.

The schizophrenia patients included in this study are in a stable phase of the disease, and having clear consciousness, communication skills, and the ability to cooperate with the investigation. Confirm that the informed consent form has been signed with the consent of the patient and their family members. Moreover, the self-assessment scale used in this study is generally filled out by patients themselves. If patients are unable to complete the questionnaire due to factors such as educational level, if necessary, the investigator can fill out the questionnaire on behalf of the patient based on their answers.

## 3 Results

**Figure 1:**
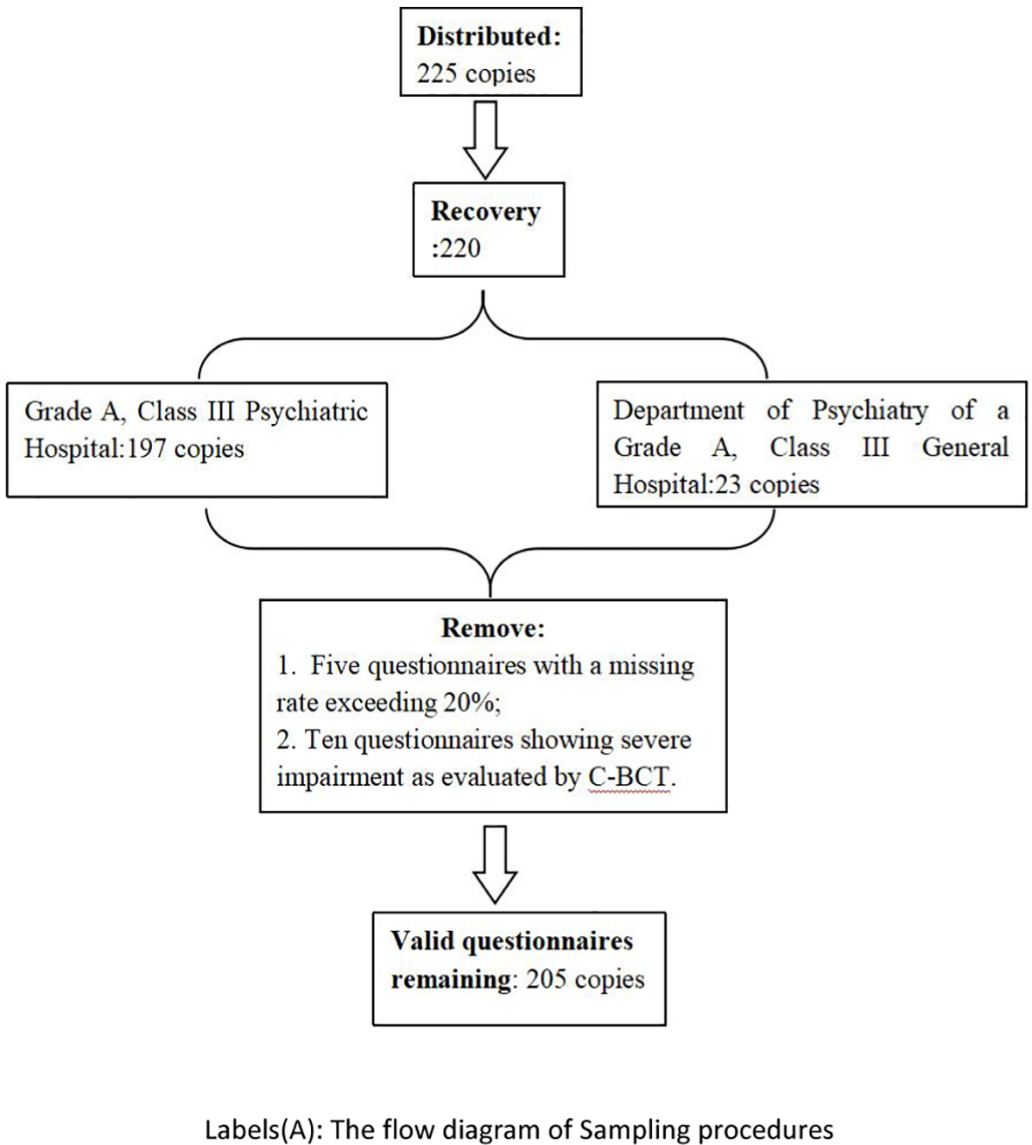
The flow diagram of Sampling procedures.

This study involved the assignment of 225 questionnaires between September 2023 and January 2024, resulting in the collection of 220 responses, comprising 197 from a Grade A, Class III Psychiatric Hospital and 23 from the Department of Psychiatry of a Grade A, Class III General Hospital, both based in Guangzhou. Five surveys with a missing rate exceeding 20% were removed, along with ten questionnaires showing severe impairment as evaluated by C-BCT. A total of 205 valid questionnaires were retained, resulting in an effective recovery rate of 91.1%. The particulars are as follow (**Figure 1**).

The level of acceptance of ACP among subjects varied. The ACP acceptance scores among patients with schizophrenia ranged from 42.00 to 106.00, with an average score of 84.76 ± 11.97. The distribution of patients in the lower middle, upper medium, and high levels of ACP acceptance was 16 (7.8%), 75 (36.6%), and 114 (55.6%) correspondingly. The overall level of acceptance of ACP was higher than the average level. The ACP attitude dimension showed that patients with schizophrenia had a maximum score of 50.00, a minimum score of 18.00, and a mean score of 37.04 ± 7.21. The ACP attitude dimension consisted of 38 patients (18.5%) in the lower medium level, 90 patients (43.9%) in the upper medium level, and 77 patients (37.6%) in the high level. The ACP attitude dimension exhibited a rather high level overall. The ACP belief component revealed that patients with schizophrenia had a maximum score of 25.00, a minimum score of 5.00, and an average score of 20.00 ± 3.00. The distribution of patients across the ACP belief dimension was as follows: 2 (1.0%) in the low level, 12 (5.9%) in the lower level, 94 (45.9%) in the upper level, and 97 (47.3%) in the high level. The total level of belief in ACP was higher than the average level. The motivation dimension of ACP revealed that the maximum score among schizophrenia patients was 35.00, the lowest score was 15.00, and the average score was 27.71 ± 4.05. Out of the total cases, 22 (10.7%) had a lower medium level of ACP motivation dimension, 105 (51.2%) had an upper medium level, and 78 (38.0%) had a high level. The overall degree of motivation in the ACP dimension was higher than the average level (**Table 1**).

**Table 1:** The level of ACP acceptance in patients with schizophrenia ( $\bar{x}\pm s$ , *n*=205).

Item	Minimum value	Maximum value	Score ( $\bar{x}\pm s$ )	Level
ACP acceptance level	48.00	106.00	84.76 ± 11.97	above-average (66-87S)
Attitude dimension	18.00	50.00	37.04 7.0.21	above-average (31-40S)
Belief dimension	5.00	25.00	20.00 0.0.00	above-average (16-20S)
Motivation dimension	16.00	35.00	27.71 7.7.05	above-average (22-29S)

The independent variables of continuous variables (age, disease duration, number of hospitalizations, and severity of psychiatric symptoms) exhibit a non-normal distribution; Moreover, age, disease duration, number of hospitalizations, and severity of psychopathological symptoms all show a non-linear relationship with ACP acceptance scores, and Pearson correlation analysis is not used. Therefore, Spearman correlation analysis is used.

In the independent variables of binary variables (gender, presence or absence of comorbidities, physical disability, personal accident, important family member illness experience, involuntary treatment experience), it was found through testing that the ACP acceptance scores under different classifications did not follow a normal distribution, and the Mann-Whitney U rank sum test was used; Among the independent variables of multiple categories (occupation, number of children, marital status, religious belief, education level, per capita monthly income of the family, medical expense payment, neurocognitive level, medication adherence level, and specific comorbidities), it was found through testing that the ACP acceptance scores under different categories did not follow a normal distribution or had uneven variances. The Kruskal-Wallis H rank sum test was used.

The correlation coefficients between acceptance of ACP and various factors including age, course of disease, number of hospitalizations, severity of psychopathological symptoms, anxiety and depression, lack of vitality, hostility and suspicion, activation, and thought disorder were found to be 0.161, 0.113, 0.235, -0.133, -0.096, -0.069, -0.004, and -0.159, respectively. Additionally, there was a correlation coefficient of -0.129. The study found that there was a correlation between age, number of hospitalizations, and activation with ACP acceptance. The data shows a substantial positive correlation between age and number of hospitalizations with ACP acceptance, as well as a significant negative correlation between activation and ACP acceptance (see **Table 2**).

**Table 2:** Univariate analysis of the acceptance degree of ACP by continuous variable data (*n*=205).

Item	ACP acceptance	
	*r*	*P*
Age	0.161^**^	0.021
Hospitalization frequency	0.235^**^	0.001
Course of disease	0.113	0.105
Severity of psychopathology symptoms	-0.133	0.056
Anxiety-depression	-0.096	0.170
Lack of energy	-0.069	0.328
Disorders of thought	-0.129	0.066
Suspicion of hostility	-0.004	0.950
Activity of activation	-0.159^*^	0.022

**P<0.01.

The subjects had a median acceptance rate of 88.00 (with a range of 80.50 to 93.00) for ACP. The median acceptance rate for ACP, regardless of comorbidities, was 2.0 (with a range of 1.0 to 2.0). The acceptance rate of ACP in patients with comorbidities (mean rank 113.76) was higher compared to those without comorbidities (mean rank 84.74) (Z=-3.386, P < 0.05). The p-value is 0.001. The median personal accident and illness experience of major family members was 1.0 (with a range of 1.0 to 1.5). The acceptance of ACP was higher among individuals with accident experience (mean rank 118.29) compared to those without accident experience (mean rank 97.94). This difference was statistically significant (Z=-2.127, P=0.033) (see **Table 3**).

**Table 3:** Univariate analysis of the acceptance of ACP by binary variable data (*n*=205).

Item	Grouping	Numberof cases	ACP acceptance		
			Mann-Whitney U	*Z*	*P*
Gender	Male	108	4900.500	-0.797	0.426
	Female	97			
Coexisting diseases	No	76	3514.500	-3.386	0.001
	Yes	129			
Physical disability	No	190	1077.500	-1.573	0.116
	Yes	15			
Unexpected experience	No	154	3147.000	-2.127	0.033
	Yes	51			
Involuntary treatment	No	43	3375.500	-0.311	0.756
	Yes	162			

There are variations in the acceptance of ACP among the three religious views, as evidenced by a significant difference (Z=7.043, P=0.030) seen through group comparisons. The level of acceptance of ACP (Attitudes toward Cultural Pluralism) toward Islam, with a mean rank of 57.00, was much lower compared to Buddhism, which had a mean rank of 143.50, and to individuals with no religious beliefs, who had a mean rank of 100.94. There were variations in the acceptance of ACP among the three levels of drug adherence, as indicated by a Z-score of 6.987 and a p-value of 0.030. The ACP’s acceptance of a high level of medication adherence (mean rank 111.26) was substantially greater than its acceptance of a medium level of medication adherence (mean rank 93.20) and a low level of medication adherence (mean rank 83.38). The acceptance of ACP varied significantly among the six specific comorbidities (Z=25.441, P < 0.001). The patients with cancer/tumor, respiratory disease, and diabetes had significantly higher acceptance of ACP compared to patients with cardiovascular disease, other diseases, and those without comorbidities. There are notable distinctions between the adoption of ACP and religious beliefs, compliance behavior and specific comorbidities (see **Table 4**).

**Table 4:** Univariate analysis of multivariate variable data on ACP acceptance (*n*=205).

Item	Group	Cases	The acceptance of ACP			
			Mean	Mean rank	*Z*	*P*
Occupation	Student	13	81.54	89.12	3.831	0.429
	Unemployed	65	83.42	99.55		
	Retire	83	86.67	110.58		
	Individual/Freedom	35	84.69	102.90		
	Career/Civil Service	9	81.67	78.50		
Marital status	Unmarried	114	84.53	142.65	2.868	0.238
	Married	64	84.17	128.18		
	Divorced/Widowed	27	87.11	147.95		
Number of children	0	141	84.90	105.26	4.389	0.111
	1	45	86.58	107.31		
	2	19	79.37	76.00		
Religious belief	None	191	84.34	100.94	7.043	0.030
	Buddhism	12	91.92	143.50		
	Islam	2	82.00	57.00		
Level of education	Primary school and below	45	84.71	95.32	5.065	0.408
	junior high school	66	84.55	101.55		
	Technical secondary school and high school	46	84.63	114.00		
	Junior college	24	86.42	106.52		
	Bachelor's degree or above	24	84.00	96.77		
Household per capita monthly income (yuan)	0-3000	49	85.73	107.10	1.889	0.596
	3001-6000	94	84.17	99.23		
	6001-9000	60	84.62	103.98		
	9001-12000	2	92.50	150.00		
Medical payment method	Self-funded	18	82.39	88.22	1.300	0.522
	Basic medical insurance for urban/rural residents	111	84.39	105.39		
	Basic medical insurance for employees/public expenses	76	85.86	103.01		
Neurocognitive level	Normal	30	83.30	134.00	4.578	0.333
	Mild impairment	31	84.32	122.37		
	Mild to moderate damage	60	85.73	141.09		
	Moderate impairment	47	83.87	148.74		
	Moderate to severe damage	37	85.84	146.24		
Level of medication adherence	Good	127	86.63	111.26	6.987	0.030
	Medium	49	83.59	93.20		
	Poor	29	78.52	83.38		
Specific comorbidities	None	76	80.83	84.74	25.441	0.000
	Cardiovascular diseases	68	88.40	116.46		
	Diabetes	18	90.50	142.00		
	Respiratory tract diseases	3	94.00	164.33		
	Cancer/Tumor	1	96.00	185.00		
	Other	39	82.41	90.29		

The statistically significant results (age, hospitalization frequency, activation, specific comorbid diseases, unexpected experience, religious belief, and level of medication adherence) were obtained through univariate analysis and were included in the candidate variables of multivariate analysis. In order to avoid missing important variables, the variables with p less than 0.2 (34) (course of disease, number of children, physical disability, severity of psychopathology symptoms, and factors) in the results of univariate analysis were included as independent variables, and the total score of ACP acceptance and scores of each dimension were taken as dependent variables. Multivariate linear regression analysis was carried out to screen variables so as to establish the optimal regression equation (see **Table 5**).

**Table 5:** Multivariate linear regression analysis of independent variableassignment method.

Independent variable	Assignment method
Age	Input in original value
Course of disease	Input in original value
Hospitalization frequency	Input in original value
Number of children	Input in original value
Specific comorbidities	
None(X1)	X1=0, X2=0, X3=0, X4=0, X5=0, X6=0(consult)
Cardiovascular diseases(X2)	X1=0, X2=1, X3=0, X4=0, X5=0, X6=0
Diabetes(X3)	X1=0, X2=0, X3=1, X4=0, X5=0, X6=0
Respiratory tract diseases(X4)	X1=0, X2=0, X3=0, X4=1, X5=0, X6=0
Cancer/Tumor(X5)	X1=0, X2=0, X3=0, X4=0, X5=1, X6=0
Other(X6)	X1=0, X2=0, X3=0, X4=0, X5=0, X6=1
Religious belief	No=1, Yes=2
Physical disability	No=1, Yes=2
Unexpected experience	No=1, Yes=2
Level of medication adherence	Good=1, Medium=2, Poor=3
Severity of psychopathology symptoms and factors	Input in original value

In the multiple linear regression model, the dependent variable is the total score of ACP acceptance. After four inputs or eliminations, two variables were included in the equation. The tolerance of each variable in the fitting equation exceeded 0.400, and the variance inflation factor (VIF) was below 2, suggesting the absence of significant multicollinearity among the variables. The model’s adjusted R2 was 0.118, with an F-value of 7.823 and a significance value of P < 0.001. This indicates that the model was statistically significant and could collectively account for 11.8% of the total variation in the equation. The data suggests that there is a positive correlation between medication compliance, hospitalization frequency, religious belief, and the severity of thinking disorder in individuals with schizophrenia, and their acceptance of ACP is higher (see **Table 6**).

**Table 6:** Multiple linear regression analysis of the influencing factors of the total score of ACP acceptance (*n*=205).

Variable	Regression coefficient	Standardized regression coefficient	*t*	*P*	*R^2^*	Adjusted *R^2^*	*F*	*P*
Constant	84.690	–	19.550	0.000	0.135	0.118	7.823	0.000
Adherence to medication	-3.503	-0.214	-3.133	0.002	–	–	–	–
Hospitalization frequency	0.377	0.192	2.840	0.005	–	–	–	–
Religious beliefs	6.746	0.171	2.538	0.012	–	–	–	–
Disorders of thought	-0.918	-0.138	-2.081	0.039	–	–	–	–

The multiple linear regression model with ACP attitude dimension as the dependent variable included two factors that were either added or removed during the analysis. The tolerance of each variable in the fitting equation exceeded 0.400, and the variance inflation factor (VIF) was below 2, suggesting the absence of significant multicollinearity among the variables. The model’s adjusted R2 was 0.059, with an F-value of 7.407 and a significance value of P=0.001. This indicates that the model was statistically significant and capable of collectively explaining 5.9% of the total variation in the equation. The study revealed a correlation between milder psychopathological symptoms in patients with schizophrenia and a higher frequency of personal accidents and illness experiences among their close relatives. This correlation was found to be associated with a more positive attitude toward participating in ACP (see **Table 7**).

**Table 7:** Multiple linear regression analysis of the influencing factors of ACP attitude dimension (*n*=205).

Variable	Regression coefficient	Standardized regression coefficient	*t*	*P*	*R^2^*	Adjusted *R^2^*	*F*	*P*
Constant	43.637	–	13.556	0.000	0.068	0.059	7.407	0.001
Severity of psychopathology symptoms	-0.329	-0.224	-3.297	0.001	–	–	–	–
Unexpected experience	2.322	0.140	2.054	0.041	–	–	–	–

The ACP faith dimension is used as the dependent variable in a multiple linear regression model. The initial input is either 1 or eliminated, resulting in a total of one variable included in the equation. The model’s adjusted R2 is 0.079, with an F value of 18.405 and a significant P value of less than 0.001. This indicates that the model is statistically significant. Furthermore, the model suggests that 7.9% of the total variation can be explained by the common equation. Additionally, the results show that patients with higher levels of schizophrenia medication compliance also have higher levels of participation in ACP beliefs (see **Table 8**).

**Table 8:** Multiple linear regression analysis of the influencing factors of ACP belief dimension (*n*=205).

Variable	Regression coefficient	Standardized regression coefficient	*t*	*P*	*R^2^*	Adjusted *R^2^*	*F*	*P*
Constant	21.799	–	46.882	0.000	0.083	0.079	18.405	0.000
Adherence to medication	-1.182	-0.288	-4.290	0.000	–	–	–	–

The multivariate linear regression model with ACP motivation dimension as the dependent variable underwent four input or removal procedures, resulting in three factors being included in the equation, as indicated in **Table 9**. The tolerance of each variable in the fitting equation exceeded 0.400, but the variance inflation factor (VIF) was below 2. This suggests that there was no evident multicollinearity among the variables in question. The model’s adjusted R2 was 0.130, with an F-value of 8.613 and a significance value of P < 0.001. This indicates that the model was statistically significant and capable of collectively explaining 13.0% of the total variation in the equation. The study revealed a positive correlation between the frequency of hospitalizations among schizophrenic patients and their level of religious belief. Additionally, it found that patients with a lesser degree of mental problem exhibited a stronger urge to engage in ACP (see **Table 9**).

**Table 9:** Multiple linear regression analysis of the influencing factors of ACP motivation dimension (*n*=205).

Variable	Regression coefficient	Standardized regression coefficient	*t*	*P*	*R^2^*	Adjusted *R^2^*	*F*	*P*
Constant	27.179	–	18.650	0.000	0.147	0.130	8.613	0.000
Hospitalization Frequency	0.154	0.231	3.446	0.001	–	–	–	–
Religious beliefs	2.446	0.183	2.735	0.007	–	–	–	–
Disorders of thought	-0.346	-0.154	-2.331	0.021	–	–	–	–

## 4 Discussion

55.6% of schizophrenia patients showed high acceptance of ACP, correlating positively with medication compliance, frequent hospitalizations, religious beliefs and inversely with thinking disorder severity. These factors should guide healthcare providers in delivering timely ACP education.

The general demographic data showed a nearly equal gender distribution, consistent with Wang Min et al’ s (35). The mean age of the patients was 50.50 ± 16.56 years, with a significant proportion (40.5%) being 60 years or older. This indicates that the population of patients with schizophrenia is experiencing an ageing phenomenon. In terms of religious beliefs, 93.2% of patients have no religious beliefs. Only 44 people (21.5%) are employed in terms of employment and livelihood, this is consistent with Chesney et al. (36). The unemployment rate is between 80% and 90%; More than half of the population (146, 53.1%) are unmarried, and the majority of households have a monthly income of 3000–6000 yuan, accounting for 45.9%, which is similar to domestic research (37), reflecting the heavy burden of schizophrenia on patients. Most hospitalized patients are middle-aged/elderly with stable symptoms but remain hospitalized due to schizophrenia’s relapsing nature. Social stigma and limited mental health awareness reduce available caregivers. Furthermore, unmarried patients often lack family support, hindering ACP. The high disability rate (38) reflects impaired self-protection ability. The long duration of illness (illness duration) and the number of hospitalizations are consistent with the results of Yang Cui’s study (39), which suggests that schizophrenia has the characteristics of long duration and is easy to recur, resulting in multiple hospitalizations and is difficult to cure. Up to 79.0% of the patients have experience of involuntary treatment, which is consistent with the research results of Pan Zhongde (40) in China, which is related to their loss of decision-making ability when the disease occurs, loss of the opportunity to express medical care, and forced to receive treatment, which is seriously against their own will, reflecting the current dilemma of mental health law. The abuse of involuntary admission cannot be avoided (41), and there is still an ethical risk of violating individual autonomy. It also suggests that patients with schizophrenia have the experience of losing their medical decision-making ability, and may have unique insights and experiences for ACP.

Additionally, it was discovered that patients with schizophrenia exhibited acceptance of ACP. The overall level is higher than average. The majority of the participants in the study were open to ACP and were eager to voice their preferences for future medical care. However, their acceptance score for ACP was slightly lower compared to patients with chronic conditions in China (87.48 ± 12.96) (42). In terms of influencing factors, chronic disease patients who adopt more positive coping strategies, have a longer course of illness, have lower levels of anxiety, and have experience taking care of dying relatives have a better acceptance of ACP. Cancer patients with high levels of education, a long course of illness, and high self-esteem have better acceptance of ACP. By comparison, it was found that similar to cancer/chronic disease studies, the more hospitalizations, the milder the cognitive impairment, and the better the ACP acceptance of schizophrenia patients. Schizophrenia patients with personal accidents or important family members who have experienced illness have a better ACP attitude.

ACP adoption has increased in China, aligning with international trends (43). Adams JR (44) discovered that a significant number of individuals suffering from mental problems express a desire to establish living wills. The purposes for conducting the analysis are as follows: (1) Schizophrenia patients, often subjected to involuntary treatment, seek participation in care planning to preserve autonomy and dignity. (2) Long-term hospitalized patients demonstrated greater treatment familiarity. (3) Symptom variability and medication side effects necessitate psychiatric management for optimal care.

ACP dimensions can be derived into the attitude dimension, belief dimension, and motivation dimension. The values for all three dimensions exceeded the average level. During the investigation, it was shown that a significant majority (up to 98%) of patients with schizophrenia were unfamiliar with terms like as ACP, living wills, and other related concepts. They had endured prolonged psychological distress. Upon receiving a thorough and comprehensive explanation, once they became aware of the potential to exercise their rights to healthcare, they demonstrated a strong inclination to engage in ACP. Instill the conviction that your dignity is safeguarded. Individuals diagnosed with schizophrenia belong to the category of vulnerable populations. Their right to health is consistently infringed due to their lack of fundamental comprehension, communication, and discernment skills (45). We must prioritize the well-being of vulnerable populations and provide adequate safeguards for patients. Treating schizophrenia is challenging, with a primary focus on improving the individual’s quality of life following recurrent episodes. Medical practitioners should strive to mitigate the symptoms experienced by patients, alleviate the discomfort resulting from the illness, and fulfill the reasonable requirements of patients for future healthcare.

This study’s results indicate a positive correlation between the frequency of hospitalizations in patients with schizophrenia and their acceptance of ACP, as well as an increased motivation to engage in ACP activities. In a recent study, Barlattani et al. demonstrates that major health crises significantly alter hospitalization rates for severe mental illnesses, including schizophrenia spectrum disorders (46). Their study revealed that while acute collective stressors may initially reduce hospitalization rates, patients with schizophrenia spectrum disorders demonstrate increased service utilization in the long term, suggesting that prolonged illness experience enhances engagement with healthcare systems. This pattern suggests that opportunities for ACP engagement may diminish during crises when hospitalization declines, yet increase during recovery phases. Consequently, proactive ACP discussions during stable periods—particularly for patients with high relapse risk—are critical to capitalize on their readiness and mitigate autonomy loss during future crises. This phenomenon is not isolated, Germany considers hospitalization experience as a significant factor (45). The disease is inherently challenging to treat, and the frequency of hospitalizations rises with the length of the illness. They possess greater familiarity with the treatment and prognosis of the disease (47) and exhibit a higher willingness to engage in diverse medical decision-making discussions compared to the initial disease (48). Furthermore, the burden of schizophrenia is heightened, and the suffering associated with the condition is intensified. In comparison to hospitalization, individuals express greater aversion to the inhumane treatment and loss of autonomy experienced during involuntary hospitalization (12). This awareness prompts proactive articulation of treatment preferences and increases ACP acceptance. Patients may be reluctant to discuss schizophrenia; however, it is evident that they harbor fears regarding involuntary hospitalization, inhumane treatment, and detrimental effects on self-esteem. These concerns can enhance patients’ adherence to medical care and motivate them to actively pursue professional treatment.

At the same time, religious beliefs among schizophrenia patients, correlate with higher ACP engagement This study encompasses the religious beliefs of Buddhism and Islam. Wang Liying (49) identified that Buddhist beliefs offer patients a religiously specific understanding of survival and death culture. This supports the scholarly view that individuals with religious beliefs tend to exhibit greater acceptance of life and death (50). This differs from research on cancer and chronic diseases in China, where traditional culture leads individuals to perceive death as a sensitive subject and to largely avoid discussions about it. Chinese Confucianism regards death as a natural part of the process of life development, fulfilling one’s life obligations in life. Death is a natural occurrence, and there is no need to worry or be worried. Chinese individuals often encounter challenges in confronting death within their cultural framework, which may influence their acceptance of ACP. Considering the impact of diverse cultural backgrounds, we can initiate discussions based on communication experiences, life values, and related topics, thereby facilitating patients’ reflections on their future medical care approaches to enhance their acceptance of ACP. Besides, patients with schizophrenia exhibit a more favorable attitude toward participating in ACP when they have experienced personal accidents and significant illnesses among family members. Yu et al. (51, 52) observed that individuals who experienced sudden personal accidents, deteriorating health periods, or illnesses of significant family members developed a more accepting attitude toward death. They exhibited less aversion to contemplating death and the future, resulting in a more positive perspective on ACP. Confucianism influences the Chinese view of life and death. Confucianism believes that death is the interest and lets nature take its course; to establish oneself in life, to never perish; life is precious, and righteousness is more important than life. When patients encounter similar experiences related to impending death or accidents, it can facilitate a serious consideration of death, fostering an attitude of acceptance rather than aversion, and encouraging preparation for death in advance. The connotation of ACP necessitates that patients engage in communication with their families. However, when significant family members become ill or pass away, patients may lose their guardians, resulting in a lack of authorized agents, which directly restricts their involvement in ACP.

This study employed the C-BCT to assess patients’ neurocognitive levels, without performing a detailed analysis of the four subtests: Trail Making Test, Digit Span, Continuous Operations, and Symbol Coding. The evaluation results indicated that 29.7% of patients exhibited normal or mild impairment, whereas 70.3% demonstrated impairment to varying degrees. This finding suggests the presence of widespread cognitive impairment, even among patients in the stable phase of the disease, aligning with current mainstream research outcomes (53). This study found a significant positive correlation between patients’ neurocognitive levels and age, course of illness, and hospitalization frequency. This aligns with Zhang Yudan’s (54) C-BCT evaluation of schizophrenia patients, indicating that older patients, those with more hospitalizations, and longer illness duration exhibit greater neurocognitive impairment. Conversely, the correlation with the severity of psychotic symptoms was relatively weak, suggesting that cognitive impairment persists throughout the disease course and does not parallel the severity of psychotic symptoms, as noted by Geng Wenbo et al. (55). This indicates that the neurocognitive level remains unchanged despite the reduction of psychotic symptoms. It is essential to focus on neurocognitive functioning and implement effective cognitive correction interventions to enhance neurocognitive abilities and facilitate recovery, while also monitoring for potential disabilities in patients. A decline in cognitive function may result in patients losing the ability to recognize their illness, impairing their decision-making capabilities, leading to denial of their condition, and consequently, refusal of medical care (28). This study identified a weak correlation between neurocognitive level and acceptance of ACP, which contradicts findings from research on ACP acceptance among patients with mild cognitive impairment in China. The influence of cognitive function decline on the acceptance of ACP may vary among patients with different diseases, and the comprehension abilities of individuals with schizophrenia cannot be evaluated solely through neurocognitive assessments. Subsequent research may validate this through a comprehensive investigation.

Furthermore, the assessment of decision-making capacity in individuals with schizophrenia is significantly complicated by the presence of neurodevelopmental comorbidities. Conditions such as Autism Spectrum Disorders (ASDs) frequently co-occur with schizophrenia. ASDs are a group of neurodevelopmental disorders characterized by persistent difficulties in social communication, restricted interests, and repetitive behaviors (56). These comorbid conditions present unique challenges to capacity assessment, including potential impairments in social communication, abstract reasoning, perspective-taking, and sensory processing sensitivities. This complexity underscores the critical need for tailored assessment approaches and potentially specialized tools when engaging in ACP discussions with patients diagnosed with schizophrenia and co-occurring neurodevelopmental disorders. Further ACP protocols could incorporate neurodevelopmentally-sensitive competence assessments, particularly for high-comorbidity subgroups.

This study indicates that in stable schizophrenia patients, psychotic symptoms may either resolve or endure over the long term, whereas prolonged medication can regulate the patient’s mental condition. The study participants were all patients in a stable phase of the disease, and their decision-making capacity, condition, and ethical considerations influenced the exploration of ACP acceptance in non-stable patients. International researchers propose that the demands and challenges of schizophrenia patients, regardless of their stability, should be acknowledged. Foreign scholars suggested that the needs and difficulties of schizophrenic patients should be listened to, whether they are in remission or not (57).

Schizophrenia symptoms can impact physical health, treatment responsiveness, and the capacity to make medical decisions. to the commencement of the sickness, their cognitive, logical, and discerning abilities are comparable to those of those without the condition; during the illness, their verbal expressions are frequently affected by the disease and do not accurately reflect their true intentions (58). In the presence of positive symptoms, the patient has auditory hallucinations and may engage in incoherent speech rather than conversing with you. Conversely, negative symptoms manifest as depression, mutism, and potential rigidity, hindering effective communication. The severity of psychopathological symptoms correlates with diminished information processing speed, impaired speech learning, and compromised social cognitive function (25). Consequently, during the stable phase of the illness, it is most appropriate to initiate ACP when the patient is alert and the intermittent symptoms are managed.

In this study, 62.0% of patients exhibited good medication adherence, marginally lower than the domestic research report of 69.1% (59); 14.1% of patients showed poor adherence, slightly below the overseas adherence rate of 30%-60% (60). The general degree of drug adherence among patients was inadequate, and they exhibited insufficient attention to adherence, potentially linked to a lack of identification with the disease (61). The study indicates that increased medication adherence among schizophrenia patients correlates with greater acceptance of ACP, enhanced belief in participation in ACP, and heightened motivation to engage in ACP. This aligns with the research findings on chronic disease ACP in China (42). Trust in medical care can enhance the efficacy of treatment, mitigate uncertainties, foster confidence in the competence of healthcare professionals, and improve adherence to medical guidance (62), hence facilitating communication between medical personnel and patients. Beverley (63) noted that the illness and its social stigma contribute to patient isolation and a deficiency of carers, culminating in delayed diagnosis and hindering patients’ access to medical care, which adversely impacts medication adherence. Studies have pointed out that long-acting injectable antipsychotics are associated with a more stable improvement in quality of life and with a good safety and tolerability profile (6). Consequently, it is imperative to enhance standardized treatment for patients, promptly restore their insight, encourage medication adherence and compliance with medical guidance, augment public awareness of mental illness, actively rehabilitate individuals with schizophrenia, eliminate stigmatization, uphold patient dignity, and advance medical care.

The R^2^ of the model obtained in this study is 0.118. It can jointly explain 11.8% of the total change in the equation. Although the model has statistical significance, there may be unmeasured information. ACP is currently in the initial stage of promotion in China. Given the unique national conditions, cultural context, and medical environment of China, the factors influencing ACP acceptance that can be included in this study are limited. Consequently, the analysis of the influencing factors presented in this study may not be sufficiently comprehensive. On the other hand, due to the limited time and energy, this study only selected two tertiary grade A hospitals in the urban area of Guangzhou, which failed to break through the influence of the region, and the representativeness of the sample was limited. This study is a cross-sectional study, which only makes a quantitative analysis of the current situation and influencing factors of ACP acceptance in patients with schizophrenia.

Future research can expand the scope of the survey to include community patients and their families. At the same time, qualitative research methods or additional interviews can be used to obtain information on participants’ subjective perspectives and thinking judgments, providing deeper explanations for the choices made by patients. In the future, when promoting the implementation of ACP in the population of schizophrenia patients, the above influencing factors can be referred to, guiding medical staff to provide timely ACP health education to patients, promoting ACP practice, and helping patients express their needs for medical care for the disease.

## 5 Conclusions

The general degree of acceptance of ACP in patients with schizophrenia was higher than average, with 55.6% of the patients showing a high level of acceptance. The ACP exhibits attitudes, beliefs, and motivation that are situated at a level of upper-middle level. Moreover, patients typically have a readiness to accept and openly express their desire for future medical care.

There is a positive correlation between medication compliance and hospitalization frequency in schizophrenic patients. Additionally, there is an inverse relationship between religious belief and thinking disorder severity. Furthermore, higher acceptance of ACP is observed in patients with fewer thinking disorders. In the future, these influencing factors can be used as a reference to guide medical staff in conducting timely ACP health education for patients with schizophrenia, integrating ACP education into long-term care protocols for patients with chronic psychiatric conditions. This will promote the practice of ACP and assist patients in expressing their medical care needs for the disease. Simultaneously, it is imperative to enhance public awareness and understanding of schizophrenia, encourage patients to proactively seek medical intervention, eradicate the social stigma associated with the illness, and empower patients to acknowledge and articulate their demands.

## Data Availability

The original contributions presented in the study are included in the article/supplementary material. Further inquiries can be directed to the corresponding authors.
