# Correction: Acceptance level and influencing factors of advance care planning among patients with schizophrenia in China: a cross-sectional survey

**DOI:** 10.3389/fpsyt.2025.1720880

**Published:** 2025-10-24

**Authors:** Shicong Liang, Lei Huang, Shu-Tong Tang, Hong-Xing Li, Jia-Wei Huang, Wen-Qing Zhou, Zhi-Chun Xia

**Affiliations:** ^1^ The Affiliated Brain Hospital, Guangzhou Medical University, Guangzhou, China; ^2^ Key Laboratory of Neurogenetics and Channelopathies of Guangdong Province and the Ministry of Education of China, Guangzhou Medical University, Guangzhou, China; ^3^ School of Nursing, Guangzhou Medical University, Guangzhou, China; ^4^ The Seventh Affiliated Hospital of Sun Yat-sen Universit, Shenzhen, China

**Keywords:** Advance Care Planning, schizophrenia, cross-sectional, influencing factors, acceptance level

The funder “Guangzhou Key Clinical Specialty (Clinical Medical Research Institute)”, to “Shicong Liang” was erroneously omitted.

The original version of this article has been updated.

